# Safe Medication Management for Polymedicated Home-Dwelling Older Adults after Hospital Discharge: A Qualitative Study of Older Adults, Informal Caregivers and Healthcare Professionals’ Perspectives

**DOI:** 10.3390/nursrep12020039

**Published:** 2022-05-31

**Authors:** Filipa Pereira, Marion Bieri, Maria Manuela Martins, María del Río Carral, Henk Verloo

**Affiliations:** 1 Institute of Biomedical Sciences Abel Salazar, University of Porto, 4050-313 Porto, Portugal; 2 School of Health Sciences, HES-SO Valais/Wallis, 1950 Sion, Switzerland; marionbieri21@gmail.com (M.B.); henk.verloo@hevs.ch (H.V.); 3 Porto Higher School of Nursing, 4200-072 Porto, Portugal; mmartins@esenf.pt; 4 Research Center for Psychology of Health, Aging and Sport Examination, Institute of Psychology, University of Lausanne, 1015 Lausanne, Switzerland; maria.delriocarral@unil.ch; 5 Service of Old Age Psychiatry, Lausanne University Hospital, 1008 Prilly, Switzerland

**Keywords:** safe medication management, home-dwelling older adults, hospital discharge, informal caregivers, interprofessional collaboration, Neuman Systems Model, stressors, reconstitution, secondary deductive content analysis

## Abstract

Safe medication management is particularly challenging among polymedicated home-dwelling older adults after hospital discharge. This study aimed to identify and categorise the stressors experienced and reconstitution strategies adopted by older adults, their informal caregivers, and healthcare professionals as they manage older adults’ medications after hospital discharge. A primary study collected the perspectives of 28 older adults, 17 informal caregivers, and 13 healthcare professionals using a qualitative descriptive design. The Neuman Systems Model was used as the basis for a secondary deductive content analysis. Findings revealed that post-discharge medication management at home involved numerous stressors, often including dysfunctions in communication, collaboration, and coordination between the multiple stakeholders involved. Reconstitution strategies for safe medication management were not always successful or satisfactory and were sometimes identified as stressors themselves. Older adults, informal caregivers, and healthcare professionals’ perspectives highlighted several potential opportunities for improving safe medication management through nurse-led, interprofessional, patient-centred practices.

## 1. Introduction

Inappropriate medication management can put older adults dependent on complex medication regimens at risk of medication-related problems (MRPs) [1,2]. These risks are increased in cases of polypharmacy (commonly defined as five or more medications daily) due to the pharmacokinetic and pharmacodynamic changes inherent in ageing [3,4]. MRPs include adverse medication reactions, medication errors, and potentially inappropriate prescriptions [2].

A quantitative descriptive study on the knowledge, attitudes, and behaviours surrounding medication use by home-dwelling older adults with chronic conditions revealed several dysfunctions that might trigger MRPs [5]: 75% of participants stated that they had received no information on medication use; 82% did not know about potential side effects; 68% did not take some medication doses; 46% discontinued their medication without asking their physician; 19% did not take their medicines regularly; 82% used other medicines without their physician’s advice; and only 7% appreciated using their medication. Inadequate explanations about medication result in omissions and incorrect dosages but can also lead to anxiety and confusion among older adults [6], increasing the risk of MRPs [7]. Nicosia et al. [8] conducted a qualitative study among older adults, primary care physicians, and pharmacists to identify MRPs from the perspective of patients and healthcare professionals. Their findings were grouped into four broad categories: obtaining medications, taking medications, medication effects, and communication and care coordination related to medications. Moreover, older adults often described MRPs with regards to their socioemotional effects on their lives, which contrasted with existing taxonomies for categorising MRPs [8].

Without preventative measures, MRPs can lead to physical and cognitive decline, exacerbated chronic medical conditions, rehospitalisations, avoidable health costs [9,10,11], and, sometimes, unplanned institutionalisation [12]. Nurses can play a crucial preventative role in ensuring safe medication management for polymedicated home-dwelling older adults [13,14]. Nursing interventions in medication management can help prevent unnecessary hospitalisations, emergency department visits, and admissions to assisted living facilities, as well as improve patients’ quality of life [15]. A recent systematic review explored nurses’ roles in medication management during transitional care, identifying three main contributions: (i) involvement in the medication reconciliation process through obtaining medication histories, performing medication reviews, and identifying medication discrepancies; (ii) involvement in interprofessional teamwork, highlighting nurses’ roles in clarifying worries about medication, interdisciplinary communication and consultation, and discharge planning and monitoring; (iii) and involvement in supporting patients, emphasising nurses’ responsibilities in communication with patients, education about medications and simplifying medication regimens, and symptoms management during transitional care [13].

In addition to nurses’ contributions, informal caregivers are also considered key partners in safe medication management for home-dwelling older adults with multiple chronic conditions, notably those who may suffer cognitive impairment or psychopathological disorders [16,17,18]. They accompany patients to health consultations, help obtain, prepare, and administer medication, monitor adherence, effectiveness, and side effects, and organise home care support [17].

Managing medication requires interprofessional, patient-centred, collaborative practices across different healthcare and social care providers, organisations, and departments [7]. Orchard [19] defined this concept as a partnership between a team of healthcare professionals and a patient, where the patient retains control over their care and has access to the team members’ knowledge and skills and thus arrives at a realistic shared plan of care with access to the resources to achieve that plan. Care coordination problems, associated with poor medication management, are even more frequent in the sensitive period of care transitions, such as discharge home from the hospital [5,6].

Different interventions have been tested to support medication management and prevent MRPs, such as pharmacist-led medication reviews [20] and nurse-led interventions to improve medication adherence [14]. Because such top-down interventions have shown limited long-term effectiveness, there is a need to consider complementary bottom-up approaches, exploring modifiable patient-centred determinants. A deeper understanding of older adults’ needs and the barriers to safe medication management might enable the development and testing of patient-centred, bottom-up interventions.

To help bridge this gap, this study aimed to use the perspectives of older adults, their informal caregivers, and healthcare professionals to identify and categorise the difficulties experienced by polymedicated home-dwelling older adults trying to manage their medication after hospital discharge (*stressors*). A second aim was to identify and categorise the response patterns adopted to overcome these difficulties and prevent MRPs (*reconstitution strategies*).

## 2. Materials and Methods

### 2.1. Study Design

This study was conducted using a qualitative descriptive design. This approach is often used in healthcare research to inform practice by providing a comprehensive, descriptive summary of the perspectives of participants directly experiencing a phenomenon [21,22]. These different perspectives were captured through semi-structured individual interviews with older adults and healthcare professionals and joint interviews with older adults and their informal caregivers. Using secondary, deductive content analysis, this study analysed data collected in a mixed-methods study supported by Switzerland’s National Research Programmes [23]. The primary study had documented the state of medication management practices among polymedicated, home-dwelling older adults after hospital discharge and had made proposals to prevent MRPs and support collaborative medication management [23,24]. Because the nature of the data collected had revealed the existence of difficulties (*stressors*) and response patterns (*reconstitution strategies*), the research team developed a secondary analysis guided by the NSM. The Consolidated Criteria for Reporting Qualitative studies (COREQ) guidelines were followed [25].

### 2.2. Theoretical Framework

The Neuman Systems Model (NSM) was used as a framework for identifying and categorising the difficulties and response patterns experienced by polymedicated home-dwelling older adults trying to manage their medication after hospital discharge and their impacts on client system stability. As per the NSM, those difficulties were considered *stressors* and response patterns were considered *reconstitution strategies* [26,27,28]. This framework also guided the study’s implications for clinical practice to support safe medication management and prevent MRPs as part of nurse-led, interprofessional, collaborative practices [29,30].

The NSM perceives the individual as a “client system”, illustrated as a series of concentric circles surrounding a basic structure [28]. The basic structure consists of factors common to all persons (such as genetic features or the strengths and weaknesses of their organ systems). The concentric circles surrounding this basic structure—the flexible line of defence, normal line of defence and lines of resistance—have a protective function for the client system’s integrity. They act as protective buffers preventing stressors from invading the client system. Stressors are described as tension-producing stimuli occurring in the client system’s internal and external environments and having the potential to cause system instability. Environmental stressors are classified as intrapersonal (when occurring inside the client system’s boundary), interpersonal (occurring outside but proximal to the client system’s boundary), and extrapersonal (occurring outside but distal to the client system’s boundary).

In the NSM, health corresponds to optimal system stability, which is “the best possible state of wellness at any given time” (p. 23). In a robust person, the flexible line of defence is strong enough to maintain system stability and prevent stressors from penetrating it. Once a stressor has overcome the normal line of defence and the lines of resistance and symptoms are being treated, the client system attempts to recover system stability. This process is called *reconstitution* and can result in higher or lower levels of system stability and wellness than before the stressor hit.

Three types of nursing interventions are proposed for retaining or returning to client system stability: primary, secondary, and tertiary interventions [28,29]. Primary preventive interventions aim to protect and reinforce the client system’s normal defences to prevent stressors from overcoming them and retaining system stability. Secondary preventive interventions aim to protect the client system’s basic structure when it has been penetrated by a stressor. Secondary interventions are essential to detecting stressors, reducing or eliminating their impact, treating symptoms, and strengthening lines of resistance. Tertiary prevention aims to protect the reconstitution of the client system’s stability after treatment/secondary prevention.

### 2.3. Participants and Recruitment

We purposefully sampled participants for recruitment with help from research nurses at the regional hospital and community healthcare centre (CHC) in the French-speaking canton of Valais, Switzerland. CHCs are patients’ first contact point with primary healthcare professionals and Switzerland’s healthcare services system. They are tasked with being patient-focused (not disease-focused) and coordinating and integrating care over time [31]. Home-dwelling older adults receive their first-line healthcare services from CHC nurses (involving case and care management, health promotion, detection, prevention, interventions, and treatments), who also play key roles in decision-making and enhancing communication and collaboration between professional and family caregivers [31,32].

Research nurses identified and recruited 28 polymedicated, home-dwelling older adults who had been discharged from hospital in the last 90 days. Ninety days was chosen because Tomlinson et al.’s systematic review and meta-analysis regarded this as a period susceptible to MRPs [33]. Older adults who met the study inclusion criteria (Table 1) had the study described to them and were asked for permission to be contacted by the researchers. Between 7 and 90 days after their discharge, investigators contacted the home-dwelling older adults by telephone and requested their consent to participate in the study. If they agreed, an initial meeting was organised in the older adult’s home in the ensuing days.

Next, an informal caregiver (*n* = 17), identified by a successfully recruited older adult as the person most involved in their medication management, was also asked for their written informed consent to participate. Informal caregivers were defined as family members, neighbours, or friends assisting dependent older adults with at least two of the basic or instrumental activities of daily living (ADLs or IADLs) or helping to ensure patient safety [34]. Our local clinical experience has shown that involvement in an older adult’s medication management involves one or more of the following activities: attending healthcare consultations, assisting with acquiring or taking medication, monitoring effectiveness and side effects, supervising adherence to medication schedules, and coordinating home care.

Older adults also identified the professional caregiver (*n* = 13) most involved in their medication management, and they were invited to participate if they were providers of community healthcare services (i.e., general practitioners (GPs), pharmacists, nurses, nursing assistants). Table 1 shows the inclusion and exclusion criteria for each group of participants, and Supplementary Figure S1 shows a diagram representing the enrolment process.

### 2.4. Data Collection

**Figure 1:**
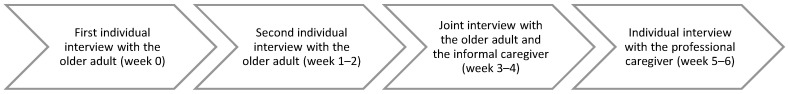
Data collection process.

Data were collected from the mixed-methods study previously mentioned [23]. That primary study used semi-structured individual and joint interviews with older adults, their informal caregivers, and healthcare professionals. Figure 1 presents the data collection process. In total, four interview guides were constructed, one for each interview, inspired from a literature review about medication management and tested in a preliminary study [35,36]. The interview guides are available in Appendix A. This section describes the collection of the parent data set.

#### 2.4.1. Home-Dwelling Older Adults

At the first meeting in the older adult’s home, the investigators described the study in detail, including its two semi-structured interviews (each lasting about an hour)—one to start immediately and the other in two to three weeks. Depending on the participant’s tiredness or their clinical condition’s complexity, sometimes only one interview was needed. Older adults deciding to participate signed a written informed consent form permitting the investigators to record pertinent personal sociodemographic, health, and comorbidity data based on the 10th revision of the International Statistical Classification of Diseases and Related Health Problems (ICD-10). They also noted numbers of ICD-10 conditions and prescribed medicines taken daily.

The initial semi-structured interview gathered older adult participants’ perspectives on their transition from the hospital to home, the treatment information given to them, possible modifications to that treatment, and whether prescribers had considered their experiences and preferences when prescribing their medications.

The second interview concentrated on patients’ medication management at home, the care network assisting their medication practices, and their lived experiences of all these interactions. Older adults were interviewed alone or, if needed, with their informal caregiver. COVID-19 health restrictions were coming into force near the end of the data collection period, resulting in the last two older adult participants and their designated healthcare professionals being interviewed by telephone.

#### 2.4.2. Informal Caregivers

When possible and appropriate, joint third interviews were organised [37] with the older adult and their informal caregiver [6], at the older adult’s home, soon after the second interview. Joint interviews enabled investigators to observe interactions concerning medication management between that dyad. Caregivers’ sociodemographic data were also collected. No informal caregivers had to be interviewed by telephone because of COVID-19 pandemic health restrictions, as the two older adults interviewed this way did not name informal caregivers involved in their medication management.

#### 2.4.3. Healthcare Professionals

Each professional caregiver participated in a roughly one-hour semi-structured interview exploring their perspectives on their interactions with home-dwelling older adults and their informal caregivers on medication management after hospital discharge. These interviews took place in professionals’ usual working environments (CHC, medical practice, or pharmacy) and during normal working hours, one to two weeks after the joint, third interview. Primary healthcare professionals’ intense workloads made arranging interview appointments more difficult. Sociodemographic and professional data were also collected.

### 2.5. Data Analyses

Participants’ sociodemographic and health characteristics were summarised and analysed using descriptive statistics. The investigators performed separate deductive content analyses of the qualitative data collected in the parent study, as per the method developed by Elo and Kyngäs [38]. Deductive content analysis was chosen because its structure was operationalised using the NSM’s theoretical framework [38]. Interviews were transcribed verbatim. Researchers read transcriptions several times to become familiar with their contents. A categorisation matrix was developed using concepts from the NSM (intrapersonal, interpersonal, and extrapersonal stressors, and reconstitution strategies). Every medication management-related quote from the parent study was coded according to these categories. Given that a structured analysis matrix was used, only aspects that fitted that matrix were chosen from the data [38]. An example of coding the data to the categorisation matrix is provided in Table 2.

Individual and joint interviews were considered equally important for capturing different perspectives of the phenomenon. In addition, data analysis was not separated by participating groups (older adults, informal caregivers, and healthcare professionals) because our focus was on the perspectives of the triad and not on each group separately. Indeed, the informal caregivers and healthcare professionals recruited were designated by the older adults.

Investigators’ data codes incorporating similar content were compared and combined into subcategories. Similar subcategories were merged by abstraction into the generic categories defined in the categorisation matrix and then separated into the main categories derived from the NSM. The investigators analysed and discussed the data until they reached a consensus. Following this data analysis, the entirety of the interview manuscripts was reviewed to validate the subcategories revealed.

### 2.6. Study Rigour

The present study’s rigour was ensured by using the four principles identified by Lincoln and Guba [39]: credibility, dependability, confirmability, and transferability.

Credibility was supported by having two researchers (MB and FP) review the data collected. Each interviewer wrote notes on the interview process to ensure that every aspect was appropriately covered as per the interview guidelines and gave a briefing before data were collected.

Confirmability was ensured by meetings to evaluate the research process and the reading and analysis of the data together as a team, by describing the participants’ demographics and by including direct quotations from them.

Dependability was ensured by defining clear study stages, keeping research diaries, having regular weekly coordination meetings, and making certain data coding was accurate.

Ensuring transferability included purposefully sampling participants according to the inclusion/exclusion criteria and providing a description of them and the context of their perceptions.

### 2.7. Ethical Considerations

The primary study was approved by the Human Research Ethics Committee of the Canton of Vaud (2018-02196, 1 February 2019) and by our study field partner’s institutional review board. All participants gave their written informed consent, and confidentiality, sociodemographic data, interview recordings, transcriptions, and files containing IDs were stored and secured using passwords. Only members of the research team have access to them.

## 3. Results

### 3.1. Sample

Following 28 interviews with older adults, no new information emerged, and we concluded that data saturation had been attained. No further participants were included. Table 3 presents the sociodemographic characteristics of our three groups of participants. Older adult patients had an average of 13 ICD-10 diagnoses when in hospital (range 3–27) and nine prescribed medications at discharge home (range 5–21). Not all the older adults designated an informal caregiver, and not every informal caregiver was involved in medication management. Indeed, only 17 participated in the study, as not all those designated agreed to do so. Thirteen of those interviewed shared the older adult’s home, three lived in the same town or village, and one lived outside the canton. Most (*n* = 11) provided daily assistance for multiple ADLs or IADLs.

Autonomy in medication management was very heterogeneous. Whereas some older adults prepared and took their medicines on their own, others needed help from their informal caregivers or health professionals (nurse or pharmacist) for medication preparation and administration. Types of help in medication management also varied and depended on stakeholders coordinating with each other. Supplementary Tables S1 and S2 provide more information on each participant.

### 3.2. Qualitative Findings

**Figure 2:**
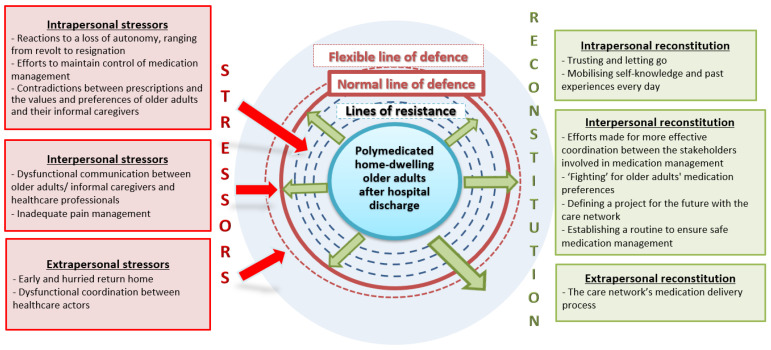
Stressors experienced and reconstitution strategies adopted by polymedicated home-dwelling older adults managing their medication after hospital discharge—inspired by the NSM [28].

The deductive content analysis of older adults, informal caregivers, and healthcare professionals’ perspectives enabled us to identify the stressors experienced by polymedicated home-dwelling older adults attempting to manage their medication after hospital discharge. We also identified the reconstitution strategies adopted to overcome these stressors, restore system stability, and optimise medication management (see Figure 2).

#### 3.2.1. Intrapersonal Stressors Affecting Safe Medication Management

Three intrapersonal stressors were identified: *reactions to a loss of autonomy, ranging from revolt to resignation; efforts to maintain control of medication management*; and *contradictions between prescriptions and the values and preferences of older adults and their informal caregivers.*

The *reactions to a loss of autonomy* were described by the triad of older adults, their informal caregivers, and healthcare professionals as the emotional and identity-related difficulties that older adults lived through with regards to their inability to perform the ADLs or IADLs independently, including medication management. Some, indeed, were quite frustrated, as older adult (OA)09 expressed:

*“Yes, because, they… I’m so used to doing everything myself that now it’s very hard not to, that is, to be just sitting here, that’s what hurts me the most.”*

Others revealed their resignation to this loss of autonomy, as OA01 noted about their hospital stay:

*“They [the hospital] did their duty, right? I couldn’t say, ‘No. I don’t want those tablets.’ Why? Because I was in their hands. I had to do what they wanted, you know?”*

This stressor revealed some older adults’ ambivalence about ‘letting go’—letting others take over—most readily seen in the implementation of home care or delegating medication management.

The stressor of *efforts to maintain control of medication management* was described by older adults and their informal caregivers, and it demonstrated older adults’ attempts to manage their daily medication despite the difficulties engendered by frequent changes to their prescriptions. About half of the older adults interviewed expressed their wish to understand their medication better, as OA22 explained:

*“I don’t want to take drugs for the sake of it. So, some people, they don’t worry. They… they swallow pills any old way. No, me, I want to know what I’m taking. And why.”*

Not being able to understand one’s new prescription is not without its consequences. Indeed, for some older adults, their lack of understanding led to non-adherence to treatment:

*“Yeah, I don’t take the Brufen^®^* [ibuprofen] *anymore. Because I think it’s [the Brufen^®^] the reason I’m in my current situation.”*

(OA03)

*“She’d given me some sleeping pills [Distraneurin^®^—clomethiazole]. Then I took a look at the box. So, it was written that, if you’ve got a cough, you shouldn’t take them. So for me, because I had asthma and I was coughing, I didn’t dare to take them.”*

(OA05)

The stressor of *the contradictions between prescriptions and the values and preferences of older adults and their informal caregivers* created tension when older adults and/or their informal caregivers were unhappy with the prescription, especially among those who self-identified as being “anti-medication”. Indeed, this can lead to resistance to treatment in opposition to the adhesion to treatment sought and recommended by healthcare professionals. This stressor was described by older adults and their informal caregivers (IC):

*“So, I said to myself, I’ll keep taking the [Atorvastatin^®^]. But as soon as I get the stent—in a little while—I’ll tell the doctor, ‘Well, I can stop that now.’ You see, I’m anti-medication.”*

(OA22)

*“(…) they changed his medications, for sure, yes, yes, clearly, and he’s got far more now, which bothered me because I’m anti-medication, but I have to get used to it.”*

(IC15)

*“No, because even in hospital, I refused to take any medications, eh? (…) I want to have some control over it.”*

(OA10)

#### 3.2.2. Interpersonal Stressors Affecting Safe Medication Management

Two interpersonal stressors affecting safe medication management were described by older adults and their informal caregivers, but not by healthcare professionals: *dysfunctional communication between older adults/informal caregivers and healthcare professionals*; and *inadequate pain management.* Regarding dysfunctional communication, inadequate explanations about changes to treatments carried out during older adults’ hospital stays were extensively evoked by the majority of the older adults and informal caregivers interviewed:

*“They could have talked to me about it before dropping it [Zoldorm^®^—zolpidem tartrate]. They don’t say much, though, eh? They don’t communicate with anyone, eh? They speak amongst themselves. They come in together; they leave together.”*

(OA20)

*“But over there, you’ve no, you’ve got no say in it. They do stuff and that’s the way it is. That’s the problem. (…) I simply obeyed.”*

(OA10)

*“So, I don’t know why they change my medications without telling me. They give you the prescription, and there you go. No, I want to know the whys and wherefores.”*

(OA22)

In addition to communication difficulties in hospital settings, communication difficulties and misaligned healthcare goals between the older adult/informal caregiver dyad and healthcare professionals were also highlighted, but far less frequently. Indeed, OA22 described an episode of conflict with her free-lance nurse when she decided—on her own initiative—to reduce the number of times per day she would check her glycaemia level:

*“(…) when I saw that I couldn’t touch anything anymore, I stopped. I only did it in the mornings. (…) So, she didn’t agree. She told me, ‘Seeing as you’re doing things your way, you don’t need me anymore,’ and she left. So, that made me a bit… I was sad. I couldn’t understand why, because me, I didn’t want to prick every one of my fingers. I wouldn’t be able to touch anything anymore.”*

(OA22)

Another interpersonal stressor was *inadequate pain management*, mentioned by four older adults and their informal caregivers. They all thought that the pain older adults suffered was insufficiently considered and that healthcare professionals could manage this better, particularly nurses, if they were more attentive to older adults’ wishes:

*“There’s the problem of pain… That might be the problem with the nurses. So, I think that, on discussing it with lots of patients, pain is rarely sorted out, after all. And then, the nurses… Well, they’re not… All in all, that’s always it. ‘Take this. The pain will pass.’ The nurses are a bit, simplistic, I’d say, in their relationship to patients and pain.”*

(OA02)

#### 3.2.3. Extrapersonal Stressors Affecting Safe Medication Management

Two extrapersonal stressors were identified: early and hurried return home and dysfunctional coordination between healthcare actors.

Many older adults and informal caregivers considered that their discharge home had occurred too early and that their recovery would have benefitted from a few more days in hospital or a rehabilitation centre. Furthermore, some informal caregivers complained that discharge was organised too rapidly, with no time to organise their care network to support the older adult’s medication management.

*Dysfunctional coordination between healthcare actors* (professional and informal caregivers) was mentioned by all three groups of participants as a stressor that affected the quality of care and medication management. It was also sometimes associated with professional constraints, such as the unavailability of nurses, the disequilibrium between the time spent on administrative tasks, and the time dedicated to the patient, or hospital or community healthcare staff turnover. This stressor also included the problems informal caregivers often faced coordinating with community care networks:

*“I’m exhausted from having to coordinate with all the different professions that don’t do things the same way—with all those people, and it’s not my field. And then, at the same time, you have to try and make a good impression. Give them a big smile. ‘How are you? Is everything all right?’ etc. And then continue making meals and so on.”*

(IC04)

From the professional perspective, one GP (Prof21) and one home-care nurse (Prof04) considered it potentially harmful for there to be a long delay between being called upon to intervene in care transitions after a hospitalisation and receiving the paperwork informing them of discharges. This was particularly relevant when there were changes in medication treatments. Prof04, a nurse in the protected accommodation where OA04 lived, described the difficulties experienced when the hospital failed to transmit new prescription and discharge documents to the GP. After OA04 had an adverse reaction to her medication, she explained:

*“I called the doctor and he said to me, ‘But I haven’t received any paperwork.’ There was no information on her… I find that there is often a lack of communication, or sometimes the hospital tells us that they are sending us the discharge documents, but then we don’t receive anything.”*

(Prof04)

To overcome these stressors and enable the reconstitution of older adults’ client systems after hospitalisation, a variety of intra-, inter-, and extrapersonal strategies were adopted.

#### 3.2.4. Intrapersonal Reconstitution Strategies

Two intrapersonal reconstitution strategies for older adults’ client systems were identified: *trusting and letting go* of medication management; and *mobilising self-knowledge and past experiences every day* in order to maintain control of one’s medication management.

After hospitalisation, one reconstitution strategy adopted by several older adults and described by all three members of their triads was to *trust others and let go* of certain tasks and responsibilities—including medication management—that they had endeavoured to control in the past. Our interviewees either sought professional help with their medication management (home care services or pharmacists) or reactivated services that they had used previously by expanding them or increasing their frequency. Confiding tasks to their care networks (healthcare professionals and informal caregivers) allowed older adults to lessen their involvement in their own medication management. This was expressed in terms of obeyance, resignation, and loss of interest but also in terms of trusting relationships.

*“She was very careful. Whereas now, she has reached a stage where she doesn’t care. I think that she has reached a stage in her life where she says to herself, ‘Well, I have no choice. I can’t manage anything anymore.’ ” (IC1a); “Yes, it’s better that they [IC] prepare it [the medication]. Otherwise, I wouldn’t be able to do it.”*

(OA01)

*“They [healthcare professionals] know more about drugs than I do.” (OA27); “We know nothing. We can’t keep up…”*

(IC27)

*“I think it suited Mrs [OA03] to have a simpler tool [pillbox] than she had had before, with big boxes, so that she didn’t have to worry about that. She just took what we had prepared. I think she trusted us too, and then they [OA03 and her husband] were very collaborative, actually.”*

(Prof03 (CHC nurse))

The second intrapersonal reconstitution strategy described by older adults, their informal caregivers, and healthcare professionals was *mobilising their knowledge and the results of their past experiences* to better organise and manage their medication day to day, especially for monitoring treatment dosages. For example, Prof07 (CHC nurse) described how OA07 took her diuretic later when she planned to go out so as not to be bothered by going to public toilets, which are not always nearby.

#### 3.2.5. Interpersonal Reconstitution Strategies

Four interpersonal reconstitution strategies were identified: *efforts made for more effective coordination between the stakeholders involved in medication management; ‘fighting’ for older adults’ medication preferences; defining a project for the future with the care network*; and *establishing a routine to ensure safe medication management*.

The efforts made by older adults and their informal caregivers to improve the effectiveness of coordination between the stakeholders involved in medication management was one of the reconstitution strategies used to stabilise older adults’ client systems.

*“Prof04 sends me the medical report with the prescription so that we have something too. And we also check with the pharmacy. So, in fact, I’m now starting to be the hub distributing things left and right. So, for each group of people, I’ve really broken down their tasks and what they’ve got to do.”*

(IC04)

In certain situations, however, if coordination reverts to dysfunction, this strategy can become a stressor too.

The strategy of *‘fighting’ for older adults’ medication preferences* sometimes implied older adults not adhering to their medical prescription. This was particularly evident with regards to the management of painkillers and insulin:

*“Yesterday, nothing. Today, nothing [Dafalgan^®^—paracetamol]. I made that decision. Why? I’ve already got a patch. It’s an analgesic patch. If I’ve got to take the Dafalgan too, I’d be amplifying those painkillers, so what’s the good of that? Just so they put me in some sort of state of… No! No, I’m not bedridden, me. Even in that state. So, I’m not taking it.”*

(OA10)

*“The Trajenta [linagliptin], that’s been since November. When I came out, they recommended taking 14 units. And me, when I saw my blood sugar level—because at home, I regulate my own blood sugar—it was still below ten. I told myself that I wasn’t going to put 14 units into me. I only put 6 units in. Because I said to myself, if I go on like this, I’ll be at under 5. And I don’t want that. I’d be scared of that.”*

(OA22)

Moreover, some older adults contested changes to their prescriptions, believing that their newly prescribed medications would be ineffective:

*“I’m not even exactly certain what the change involved. It was stuff for sleeping that they took away from me, and they’d given me something else. But I wasn’t sleeping with that. So, I telephoned my GP, and the CHC’s head nurse to explain it to her, to ask if I could have the Dalmadorm^®^ [flurazepam] back again. I don’t know. I can sleep with that, whereas with the other one, I had to wait two hours before falling asleep.”*

(OA20)

The strategy of *defining a project for the future with the care network*—one with the older adult’s preferences clearly established—seemed to facilitate coordination between the different stakeholders involved in medication management. This strategy was described by all three types of participants.

*“And it’s true that with OA03 we had, huh, we were on the same wavelength. You see? We told her what she wanted to hear—and that was that she could stay at home longer, because that’s what she wanted. And that we’d be helping her with that; that we’d be putting things in place [about medication management]. It was really so she could stay at home, and we were going to do everything possible. As a result, she agreed to lots of things.”*

(Prof03 (nurse))

The reconstitution strategy of *establishing a routine to ensure safe medication management* helped to put in place sustainable habits, almost becoming part of older adults’ daily traditions or rituals—this often involved ways of storing and taking medications day to day. Indeed, the older adults interviewed wanted the minimum possible changes to their medication regimens. However, they were open to experimenting with approaches or tools that made things safer for them (such as new home care services or new types of pillbox).

#### 3.2.6. Extrapersonal Reconstitution

Extrapersonal interventions helping with reconstitution all involved *the care network’s medication delivery processes* and were described by all three groups of the triad. Our findings showed that these included several components: prescribing, transcribing, dispensing, administering, and monitoring. Nurses were particularly involved in the transcription and dispensing phases with home-dwelling older adults and sometimes with the administration phase too. Their role in monitoring was less explicit, as shown by some difficulties in interpreting clinical signs/symptoms and transmitting information to the rest of the interdisciplinary team. These reconstitution strategies, therefore, had the potential to become stressors at some point.

*“And they [CHC nurses] are very nice and everything, but after they’ve written heaps and heaps with a special pen, it’s recorded back there. But it all stays back there. I said, ‘But when something happens like last time, what good does it do to have it all back there? You should send it to my GP. That’s the least you could do.’ I always thought that’s what they did with all those reports. So, I said, ‘Listen here. It’s essential that when something special happens, like last time, when you come by on Tuesday morning and I have to go to the emergency department in the afternoon. You note it all down, you write chapters…’ It’s not normal that the doctor’s not informed. I had to tell her myself.”*

(OA28)

Because these stressors and reconciliation strategies were not always identified by all three groups of participants (older adults, informal caregivers, and healthcare professionals), Table 4 presents who described them.

## 4. Discussion

Exploring the perspectives of older adults, their informal caregivers, and healthcare professionals allowed us to identify the difficulties experienced by polymedicated home-dwelling older adults trying to manage their medication after hospital discharge and to categorise them as stressors within the NSM. Our findings revealed that these older adults were particularly exposed to a variety of intra-, inter-, and extrapersonal stressors that affected their medication management. We also observed that response patterns—or reconstitution strategies—for ensuring safe medication management were not always successful or satisfactory. Indeed, some participants’ reconstitution strategies were identified as stressors as well.

The NSM contributed to describing and understanding these difficulties (stressors) and their response patterns (reconstitution strategies). Prior to recruitment, our older adult participants were exposed to negative stressors that disrupted their lines of defence, destabilised their client system, and pushed them towards hospitalisation. Hospital admission led them towards the reconstitution process through secondary prevention measures that required the client system to alter itself in order to preserve and protect its basic structure. Discharge home occurred when some degree of system stability had been reached. However, in our study, this degree of stability was not perceived from the point of view of older adults and their informal caregivers (the extrapersonal stressor of “early and hurried return home”). According to the NSM, reconstitution after hospital discharge depends upon a successful mobilisation of the older adult’s resources to prevent further regression or reaction to stressors.

There is a greater risk of MRPs at hospital discharge because the client system is less well protected by its recently disrupted lines of defence—there is a state of entropy where older adults have less energy available than they need [28]. Thus, when one or more stressors related to medication management cross the lines of defence, the instability triggered in the client system could result in MRPs and adverse health outcomes.

Our findings revealed that the stressors on safe medication management often concerned dysfunctions in the communication, collaboration, and coordination between the multiple stakeholders involved in it. This was concordant with the findings of Nicosia, Spar [8] about MRPs as seen from patients’ and healthcare professionals’ perspectives. Their participants identified communication and care coordination related to medication as an MRP; they also identified insufficient information about medications. The latter, however, was not corroborated by our findings, which revealed that older adults did not necessarily want to know more about their medication but did want to be properly informed of any changes and participate in decisions. The other MRPs described in the Nicosia, Spar [8] study—obtaining medications (e.g., problems of cost and insurance coverage), taking medications (e.g., organisation and remembering to take pills), and medication effects (including side effects and concerns about effectiveness)—were not identified by our participants as stressors on safe medication management. This might be explained by differences in the studies’ inclusion criteria. The older adults in the Nicosia, Spar [8] study were not necessarily polymedicated (they were taking at least one medication) and had not been discharged from hospital. Furthermore, the clinical situations in our study were more complex: older adults were receiving considerable support for certain medication-related activities, such as obtaining medications or organising and taking medications, either from informal caregivers, healthcare professionals, or even both. For example, the majority of older adults in our study had a pillbox prepared by the CHC’s nurse or the community pharmacy, and their informal caregiver reminded them when to take their medication. In relation to medication effects, the older adults and their informal caregivers in our study described their trust in healthcare professionals. Because they trusted them, they considered the prescribed medications to be appropriate. Trust was described by the members of all three groups and was categorised within the reconstitution strategy of “trusting and letting go”.

Despite older adults and their informal caregivers frequently evoking the trust they placed in their care network, our findings showed that they were rarely integrated into decision-making about returns home, discharge planning, or changes to medication. Care networks’ decisions concerning older adults failed to consider their values and preferences, as evidenced in other studies [40,41]. Indeed, according to our findings, older adults were the recipients of care rather than partners in the care networks involved in their medication management. This was in line with Holmqvist et al.’s [42] findings about home-dwelling older adults’ experiences of medication-use evaluations. Even though older adults trusted that their physicians would regularly undertake medication-use evaluations and they were willing to be actively involved in it, that did not always occur, potentially contributing to an increased risk of MRPs.

Our findings showed that not being involved in medication-related decisions introduced two potential concerns into older adults’ medication management. One was the wish to be ‘in control’, an attitude that was sometimes responsible for patient discord with healthcare professionals and deliberate non-adherence to treatment. The second attitude was one of ‘letting go’, passively obeying instructions, losing interest, participating less, and handing over full control of medication management to the care network. Letting go contributed to older adults’ difficulties understanding their medication regimens and the changes made to them. We also noted that older adults who no longer thoroughly understood their medication situation reported trusting more and participating less.

These findings, however, could not tell us whether older adults ‘let go’ because they trusted in their care network or whether they learned to trust their care network because they had had to ‘let go’ (due to problems of comprehension or health literacy). Whatever the case, our findings revealed the paradox of the mutual trust between patients and professional caregivers (the foundation of any therapeutic relationship) and lessening participation and understanding: older adults should benefit from that trust by being given better information and being allowed to participate more actively in decision-making concerning them [43,44].

Although the data collected from older adults and their informal caregivers allowed us to identify and categorise three types of stressors and reconstitution processes, professional perspectives remained focused on extrapersonal stressors and extrapersonal reconstitution. This was congruent with the findings of Nicosia et al. [8], in which older adults described MRPs from the socioemotional effects they had on their lives, in contrast to professional taxonomies for categorising MRPs (adverse medication reactions, medication errors, and potentially inappropriate prescriptions). These misaligned perspectives between older adults/informal caregivers and their healthcare professionals may have revealed a misalignment of their medication management goals and values and a failure of patient-centeredness. However, in a shared-decision making approach, patients should be considered pro-active partners and co-decision-makers [40,41]. When they are not, the risks of MRPs, such as non-adherence to treatment or medication errors, are higher [45,46]. A systematic review about the relationship between patient empowerment and medication adherence suggested that patient empowerment could promote medication adherence, but this required a co-constructed feeling of control in the professional caregiver–patient dyad induced by a “joint empowerment” approach [47].

Our findings suggested that older adults’ medication management after hospital discharge could benefit from a more nurse-led, empowering approach, with nurses sharing information on best practices in medication management and agreeing on responsibilities with older adults and their informal caregivers. Although patient empowerment has been promoted for safe medication management during hospital transitions [33], our results showed that this practice was not yet sufficiently implemented in practice among polymedicated home-dwelling older adults.

Because our older adult participants’ lines of defence had recently been disrupted and they presented with a greater risk of MRPs, nurse-led, interprofessional, collaborative practices could have helped to strengthen those lines of defence through secondary and tertiary prevention interventions. According to the *NSM Perspective of Nurse-Led Interprofessional Collaborative Practice* developed by Montano [30], nurses can clarify and share decision-making processes and facilitate discussions between interprofessional caregivers and older adults. The role of nurses in medication management during care transitions include: (i) medication reconciliation (obtaining medication histories, performing medication reviews, identifying medication discrepancies); (ii) collaboration with other healthcare providers in clarifying medication concerns, interdisciplinary communication and consultation, and discharge planning and monitoring; and (iii) provision of support to healthcare recipients, as professionals responsible for interpersonal communication with patients, medication education, the simplification of medication regimens, and symptoms management during transitional care [13]. Since the stressors on medication management and the reconstitution strategies identified for dealing with them mostly involved communication and coordination issues, our findings suggested that nurses’ responsibilities in medication management should be better defined, especially in hospitals, during care transitions, and in community healthcare settings after hospital discharge.

Furthermore, polymedicated, home-dwelling older adults at a high risk of MRPs should systematically be assigned a geriatric community-care nurse manager from a nearby CHC. Among a nurse manager’s tasks are fulfilling patients’ needs regarding medication management and preventing MRPs [48]. Using an interprofessional, evidence-based approach, co-constructed with older adults and informal caregivers, community-care nurse managers could contribute significantly to preventing hospital admissions, readmissions, institutionalisation in long-term care facilities, and early death, thus also helping to limit costs to the healthcare system [49].

### Study Strengths and Weaknesses

The present paper provides a timely contribution to the growing body of evidence offering multiple perspectives on the difficulties, response patterns, and nurse-led, interprofessional, collaborative practices surrounding safe medication management for home-dwelling older adults after hospital discharge. The paper also introduces a categorisation of those difficulties into different types of stressors and of those response patterns into different types of reconstitution strategies, all within a globally recognised nursing model for community healthcare. Moreover, performing a secondary content analysis allowed us to take advantage of and benefit from existing research data to find answers to a question that was not asked in the primary study [49]. In the current trend towards open data, which encourages the repurposing of research information, this study’s design was a good example of how to transpose existing data to a nursing model’s perspective.

The study had some limitations. Although we considered the secondary analysis to be a strength, the interview guides were constructed based on a literature review on medication management, not the NSM; however, they did follow the philosophy of the NSM. We believe a data collection approach inspired directly by the NSM would have provided more exhaustive information, especially regarding extrapersonal stressors and reconstitution strategies. Secondly, in March 2020, data collection halted due to the risks that the COVID-19 pandemic posed to older adults and their often relatively old informal caregivers. Data collection also ceased with community healthcare professionals as their time and skills were in great demand during this health crisis. One limitation of our interviews was the difficulty maintaining older adults’ and their informal caregivers’ focus on medication management and not on other ADLs or IADLs. Maintaining a focus on the older adult’s most recent care transition and ignoring earlier hospitalisations was also difficult. It is also possible, despite our best efforts at methodological rigour, that some of our participants’ answers were influenced by social desirability bias and that their medication management was less effective than described in the interviews. Finally, despite our study’s multiple perspectives on safe medication management, our participants were limited to one Swiss canton. Drawing conclusions for other cantons and countries should be done with care.

## 5. Conclusions

This paper provides a useful and original standpoint regarding bottom-up nursing approaches to improve safe medication management among polymedicated, home-dwelling older adults with multiple chronic conditions. Older adults, informal caregivers, and healthcare professionals’ perspectives revealed several potential opportunities for improving safe medication management through nurse-led, interprofessional, collaborative, patient-centred practices. The majority of older adults wish to be and have the capacity for being actively involved in their medication management. Their willingness to participate may represent a poorly used resource in medication management processes. Polymedicated, home-dwelling older adults might benefit significantly from an approach that empowers their participation in safe medication management. Nurses should be encouraged to share information on best practices on that topic with older adults and their informal caregivers, and care networks should agree on sharing responsibilities with them.

## Figures and Tables

**Table 1:** Inclusion and exclusion criteria for different groups of participants.

Participants	Inclusion Criteria	Exclusion Criteria
Older adults	Aged 65 or above Hospitalised within the last 90 days [33] Managing at least five different medications daily	Unable to speak and understand French
Informal caregivers	Aged 18 or above Designated by the older adult as the most significant informal caregiver involved in their medication management	Unable to speak and understand French
Healthcare professionals	Designated by the older adult as playing a key role in their medication management	Student Apprentice Unable to speak and understand French

**Table 2:** An example of coding the data to the categorisation matrix through deductive content analysis within the NSM.

Participants	Intrapersonal	Interpersonal	Extrapersonal
Stressors experienced in medication management after hospital discharge	“I don’t take the Brufen^®^ [Ibuprofen] anymore. Because I think it’s [the Brufen^®^] the reason I’m in my current situation.” (OA03)	“I don’t know why they change my medication without telling me.” (OA22)	“Too fast. Yes, yes. I should have stayed a week longer.” (OA09)

**Table 3:** Participants’ sociodemographic and professional characteristics and older adults’ numbers of prescribed medications and ICD-10 diagnoses.

Sociodemographic and Professional Characteristics	Older Adults (*n* = 28)	Informal Caregivers (*n* = 17)	Healthcare Professionals (*n* = 13)
Sex (number)			
Female	11	15	10
Male	17	2	3
Age (years)			
Mean/median	81/83	68/67	44/45
Range	66–94	48–86	28–58
Relationship with the older adult			
Spouse/partner		10	
Child		6	
Daughter-in-law		1	
Profession (number)			
Retired	28	9	-
Employed	0	7	13
Unemployed	0	1	-
Nurse			5
Pharmacist/Pharmacy Assistant			4
General Practitioner/Specialist			4
ICD-10 diagnoses (number)			
Mean/median	13/12		
Range	3–27		
Medications (number)			
Mean/median	9/8		
Range	5–21		

**Table 4:** Contributions to identifying and describing stressors and reconstitution strategies made by each participant group.

Stressors and Reconstitution Strategies Identified		Described by
Intrapersonal stressors	Reactions to a loss of autonomy, ranging from revolt to resignation	Older adults Informal caregivers Healthcare professionals
	Efforts to maintain control of medication management	Older adults Informal caregivers
	Contradictions between prescriptions and the values and preferences of older adults and their informal caregivers	Older adults Informal caregivers
Interpersonal stressors	Dysfunctional communication between older adults/informal caregivers and healthcare professionals	Older adults Informal caregivers
	Inadequate pain management	Older adults Informal caregivers
Extrapersonal stressors	Early and hurried return home	Older adults Informal caregivers
	Dysfunctional coordination between healthcare actors	Older adults Informal caregivers Healthcare professionals
Intrapersonal reconstitution	Trusting and letting go	Older adults Informal caregivers
	Mobilising self-knowledge and past experiences every day	Older adults Informal caregivers Healthcare professionals
Interpersonal reconstitution	Efforts made for more effective coordination between the stakeholders involved in medication management	Older adults Informal caregivers
	‘Fighting’ for older adults’ medication preferences	Older adults Informal caregivers
	Defining a project for the future with the care network	Older adults Informal caregivers Healthcare professionals
	Establishing a routine to ensure safe medication management	Older adults Informal caregivers
Extrapersonal reconstitution	The care network’s medication delivery process	Older adults Informal caregivers Healthcare professionals

## Data Availability

The data are available upon reasonable request.

## Supplementary Materials

The following supporting information can be downloaded at: https://www.mdpi.com/article/10.3390/nursrep12020039/s1. Figure S1: Enrolment Process; Table S1: Older Adult Participants’ Characteristics; Table S2: Informal caregivers and healthcare professionals’ characteristics.

## Appendix A. Interview Guides

The interview guides from the primary study were inspired by our literature review and tested in a preliminary study [35,36]. The original French versions were approved by the Human Research Ethics Committee of the Canton of Vaud on 5 July 2017 (2017-01025) and 1 February 2019 (2018-02196).

**Table A1:** Interview guide 1 with the older adult.

Topics	Items
(1) Presentation	Presentation of the study Presentation of the interview’s objectives Details of the ethics measures taken
(2) Experience of hospitalisation and hospital discharge	General experience of the hospital stay Experience with medication received in hospital: Changes to usual treatment in hospital Information received in hospital about medication changes People involved Tools received in hospital to help manage medication at home
(3) Experience of the return home	General experience and process of return home Experience with medication since returning home: Management of medicines (and any changes made in hospital) since return home People involved in medication management at home Perceptions/experiences of taking several medications per day Methods put in place to avoid forgetting to take medication, keep to the right schedule and avoid taking the wrong medication Taking other health or wellness products
(4) Socio-demographic data	
(5) End of the interview	Reminder of the ethical requirements for using the data collected in the interview

**Table A2:** Interview guide 2 with the older adult.

Topics	Items
(1) Presentation	Presentation of the interview’s objectives Reminder of the details of the ethics measures taken
(2) Daily medication management	Description of daily medication management: Medication management locations (taking, storing) Schedules Routines
(3) Support at home for medication management	People involved in the day-to-day management of medicines Who (people involved) Frequency of assistance Type of assistance
(4) Experiences with medication	Medication habits and changes: Time of onset of medication (before, during and after hospitalisation) Knowledge about medications: Indication for each medication Effects of each medication Possible precautions Most important medication Satisfaction with information received about each medication Wish to ask questions about a particular medication
(5) End of the interview	Reminder of the ethical requirements for using the data collected in the interview

**Table A3:** Joint interview with the older adult and their informal caregiver.

Topics	Items
(1) Presentation	Presentation of the study Presentation of the interview’s objectives Details of the ethics measures taken
(2) Older adult–informal caregiver relationship	The relationship between them Assistance provided in ADL and IADL
(3) Experience of the return home	Process of hospital discharge and return home: Experience with medication changes Information received Experience with medication since returning home Support for medication management
(4) Involvement in medication management	Activities where the informal caregiver is involved in medication management How it happens Example
(5) Sociodemographic data	
(6) End of the interview	Reminder of the ethical requirements for using the data collected in the interview

**Table A4:** Professional caregiver involved in medication management.

Topics	Items
(1) Presentation	Presentation of the study Presentation of the interview’s objectives Details of the ethics measures taken
(2) Experience with the older adult in relation to medication management	Since when has Mr/Mrs X been followed Type(s) of medication management intervention (prescription, preparation, administration, monitoring, etc.) Frequency of intervention Process of hospital discharge and the return home: Information received before the first visit after hospitalisation Possible changes in usual medication Progress of the medication adjustment Any difficulties encountered with Mr/Mrs X regarding medication management
(3) Sociodemographic and professional data	
(4) End of the interview	Reminder of the ethical requirements for using the data collected in the interview
