# Supplementary material for: Safe Medication Management for Polymedicated Home-Dwelling Older Adults after Hospital Discharge: A Qualitative Study of Older Adults, Informal Caregivers and Healthcare Professionals’ Perspectives
Source: Nurs Rep. 2022 May 31;12(2):403–23. doi: 10.3390/nursrep12020039 (PMC9230543; doi:10.3390/nursrep12020039)
Supplement: Supplementary file 1 [file nursrep-12-00039-s001.zip › Supplementary Table S1_PoP.pdf]

Supplementary Table S1. Older participants' characteristics.

Older adult ID	Age	Sex	Marital status	Length of hospital stay (days)	Number ICD-10	Number of medications	Principal ICD-10 diagnosis for hospitalisation
**OA01**	92	F	widow	6	7	12	M80.88 Other osteoporosis with current pathological fracture, vertebra(e)
**OA02**	66	M	married	112	27	21	B25.80 Cytomegaloviral disease of the digestive system
**OA03**	73	F	married	39	12	8	C16.3 Malignant tumour: pyloric antrum
**OA04**	82	F	divorced	20	25	14	S70.0 Contusion of hip
**OA05**	71	F	widow	60	12	6	K92.1 Melaena
**OA06**	86	M	married	14	10	9	J18.0 Bronchopneumonia, unspecified
**OA07**	94	F	widow	12	10	8	I50.01 Right ventricular failure (secondary to left heart failure)
**OA08**	73	F	widow	12	3	10	N30.0 Acute cystitis
**OA09**	83	F	widow	37	14	8	T84.04 Mechanical complication of internal joint prosthesis: hip joint
**OA10**	85	M	widower	18	18	7	M80.98 Unspecified osteoporosis with pathological fracture
**OA11**	82	M	married	41	18	13	D64.8 Other specified anaemias
**OA12**	82	M	widower	14	17	8	I50.01 Right ventricular failure (secondary to left heart failure)
**OA13**	75	M	widower	14	9	7	I63.0 Cerebral infarction due to thrombosis of precerebral arteries
**OA14**	88	F	widow	17	10	7	S22.4 Multiple fractures of ribs

**OA15**	85	M	married	34	16	13	I63.4 Cerebral infarction due to embolism of cerebral arteries
**OA16**	*Recruited but not polymedicated at the time of the interview*						
**OA17**	87	M	married	47	22	11	K80.30 Calculus of bile duct with cholangitis
**OA18**	86	M	married	13	5	5	I63.8 Other cerebral infarction
**OA19**	84	M	married	5	9	6	G45.0 Vertebrobasilar artery syndrome
**OA20**	69	M	divorced	13	14	13	I74.3 Embolism and thrombosis of arteries of lower extremities
**OA21**	75	F	married	13	16	6	R26.8 Other and unspecified abnormalities of gait and mobility
**OA22**	83	F	widow	8	5	7	J13 Pneumonia due to Streptococcus pneumoniae
**OA23**	87	F	widow	5	8	11	A08.1 Acute gastroenteropathy due to Norwalk agent
**OA24**	74	M	married	8	13	9	J18.1 Lobar pneumonia, unspecified
**OA25**	88	M	married	12	18	7	I50.01 Left ventricular failure
**OA26**	76	M	married	1	4	4	C22.0 Liver cell carcinoma
**OA27**	77	M	married	12	11	8	C20 Malignant neoplasm of rectum
**OA28**	89	M	widower	14	9	14	K56.5 Intestinal adhesions [bands] with obstruction (post-infection)
**OA29**	81	M	married	4	9	8	K80.20 Calculus of gallbladder without cholecystitis without obstruction
