# Supplementary material for: Safe Medication Management for Polymedicated Home-Dwelling Older Adults after Hospital Discharge: A Qualitative Study of Older Adults, Informal Caregivers and Healthcare Professionals’ Perspectives
Source: Nurs Rep. 2022 May 31;12(2):403–23. doi: 10.3390/nursrep12020039 (PMC9230543; doi:10.3390/nursrep12020039)
Supplement: Supplementary file 1 [file nursrep-12-00039-s001.zip › Supplementary Table S2_PoP.pdf]

Supplementary Table S2: Informal and professional caregivers' characteristics.

Older adult ID	Informal caregiver ID	Age	Sex	Relationship	Healthcare Professional ID	Profession	Age	Sex
**OA01**	IC01a	59	F	Daughter	Prof01a	Pharmacist	40	F
	IC01b	67	F	Daughter	Prof01b	Pharmacy assistant	28	F
**OA02**	IC02	58	F	Wife	No designated healthcare professional			
**OA03**	IC03	71	M	Husband	Prof03	Nurse	30	F
**OA04**	IC04	54	F	Daughter	Prof04	Nurse	43	F
**OA05**	Not involved in medication management				No designated healthcare professional			
**OA06**	Refused to participate				No designated healthcare professional			
**OA07**	IC07	52	F	Daughter-in-law	Prof07	Nurse	31	M
**OA08**	IC08	55	F	Daughter	Prof08	Pharmacist	56	F
**OA09**	Not involved in medication management				No designated healthcare professional			
**OA10**	Not involved in medication management				Refused to participate			
**OA11**	IC11	57	F	Wife	Refused to participate			
**OA12**	IC12	48	F	Daughter	No designated healthcare professional			
**OA13**	Not involved in medication management				Refused to participate			

**OA14**	Refused to participate				No designated healthcare professional			
**OA15**	IC15	84	F	Wife	Prof15	Nurse	58	F
**OA17**	IC17a	85	F	Wife	Prof17	GP	54	F
	IC17b	52	F	Daughter				
**OA18**	IC18	86	F	Wife	Prof18	Pharmacist	54	M
**OA19**	Refused to participate				No designated healthcare professional			
**OA20**	Refused to participate				Prof20	Nurse	46	F
**OA21**	IC21	80	M	Husband	Prof21	GP	39	M
**OA22**	Not involved in medication management				Refused to participate			
**OA23**	Not involved in medication management				No designated healthcare professional			
**OA24**	Not involved in medication management				Refused to participate			
**OA25**	IC25	76	F	Wife	Unreachable			
**OA26**	IC26	75	F	Wife	No designated healthcare professional			
**OA27**	IC27	80	F	Wife	Unreachable			
**OA28**	Not involved in medication management				Prof28	GP	?	F
**OA29**	Not involved in medication management				Prof29	Oncologist	47	F
