# Supplementary figures and images for: Safe Medication Management for Polymedicated Home-Dwelling Older Adults after Hospital Discharge: A Qualitative Study of Older Adults, Informal Caregivers and Healthcare Professionals’ Perspectives
Source: Nurs Rep. 2022 May 31;12(2):403–23. doi: 10.3390/nursrep12020039 (PMC9230543; doi:10.3390/nursrep12020039)

**Supplementary Figure S1.** Description of the enrolment process.

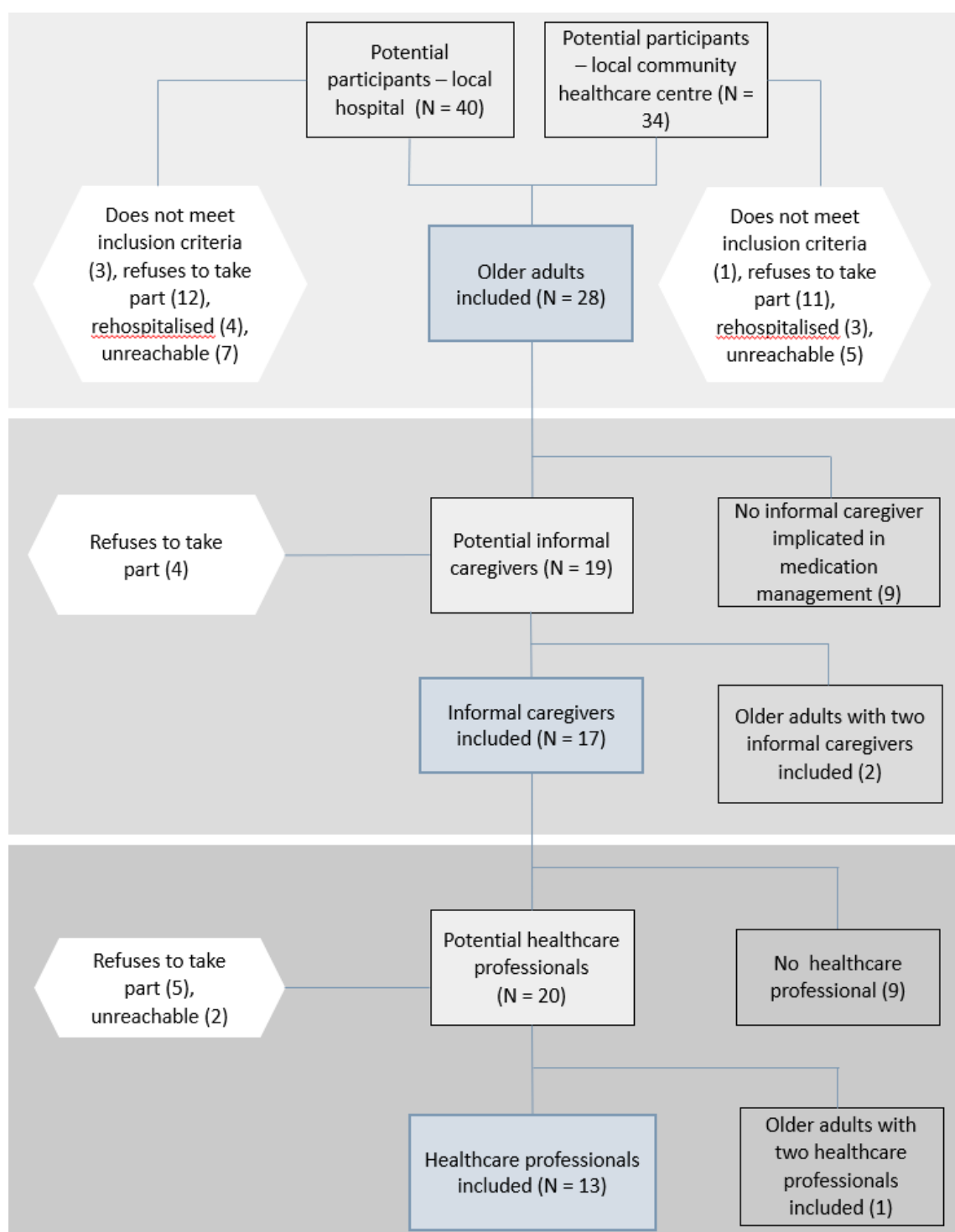

Supplement: Supplementary file 1 [file nursrep-12-00039-s001.zip › Supplementary Figure S1_PoP.pdf]
